# CHD1 and CHD2 are positive regulators of HIV-1 gene expression

**DOI:** 10.1186/1743-422X-11-180

**Published:** 2014-10-08

**Authors:** Melissa J Rodgers, David J Banks, Kenneth A Bradley, John AT Young

**Affiliations:** The Nomis Center for Immunobiology and Microbial Pathogenesis, The Salk Institute for Biological Studies, 10010 North Torrey Pines Road, La Jolla, CA 92037 USA; Department of Microbiology, Immunology, and Molecular Genetics, University of California, Los Angeles, CA 90095 USA

**Keywords:** HIV, CHD1, CHD2, Chromatin remodeling, Transcription

## Abstract

**Background:**

Retroviruses encode a very limited number of proteins and therefore must exploit a wide variety of host proteins for completion of their lifecycle.

**Methods:**

We performed an insertional mutagenesis screen to identify novel cellular regulators of retroviral replication.

**Results:**

This approach identified the ATP-dependent chromatin remodeler, chromodomain helicase DNA-binding protein 2 (CHD2), as well as the highly related CHD1 protein, as positive regulators of both MLV and HIV-1 replication in rodent and human cells. RNAi knockdown of either CHD2 or the related CHD1 protein, in human cells resulted in a block to infection by HIV-1, specifically at the level of transcription.

**Conclusions:**

These results demonstrate that CHD1 and CHD2 can act as positive regulators of HIV-1 gene expression.

## Background

Retroviruses cause a wide range of diseases in humans and animals, including cancer (HTLV-1, and HTLV-2) and AIDS (HIV-1 and HIV-2) in humans. These viruses exploit a large number of cellular proteins and other factors to facilitate different steps of virus replication. The early stages of retrovirus replication involve virus-receptor and -co-receptor interactions, membrane fusion, cytosolic entry, reverse transcription, nuclear import and integration of the viral genome into a host chromosome to form the provirus. The late stages involve proviral transcription, viral RNA splicing, polyadenylation and cytoplasmic transport, protein translation, and viral assembly, budding and maturation.

A number of comprehensive genetic screens, most relying on RNAi-knockdown technology, have revealed a number of cellular factors that are important for replication by retroviruses such as HIV-1 [1–4]. While this is a powerful approach, its impact is limited by the fact that the RNAi-knockdown level seen with individual genes is often incomplete, i.e. expression of the target gene is reduced but is not completely ablated. To overcome this potential limitation of this technology, we are adopting complementary approaches including insertional mutagenesis-based screening. This approach has revealed important regulators of retroviral replication that were missed by siRNA screening, including ZASC1, a key regulator of HIV-1 transcriptional elongation [5, 6] and the cellular sulfonation pathway that also regulates retroviral gene expression [7].

Here we describe results of another insertional mutagenesis screen that have revealed CHD2, and the related CHD1 protein, as positive regulators of HIV-1 gene expression. These proteins are members of the chromodomain helicase DNA binding protein (CHD) family of ATP-dependent chromatin remodelers, which are important regulators of chromatin structure and transcription [8]. Currently there are four distinct families of ATP-dependent chromatin remodelers, including the SWI/SNF, ISWI, INO80 and CHD families, and all are characterized by the presence of a SWI2/SNF2-family ATPase domain [9]. These chromatin remodelers utilize the energy of ATP hydrolysis to move, eject or restructure nucleosomes, thereby controlling the access of regulatory proteins to DNA or histones [10]. The CHD family of proteins is distinguished by the presence of two tandem chromodomains located in the N-terminal region of the protein in addition to the centrally located SWI2/SNF2-family ATPase domain [11]. Although budding yeast have a single CHD1 protein, mammals express nine different CHD proteins [11].

Currently, CHD1 is the better characterized of these two proteins. CHD1 localizes to regions of active transcription and interacts with transcription elongation complexes including the PAF and FACT complexes [12–15]. The yeast CHD1 protein can exist as a monomer or a dimer [16, 17], and is an essential component of the yeast SAGA and SLIK HAT complexes [16]. The chromodomains of human CHD1 can specifically bind methylated H3K4 suggesting a possible mechanism for targeting the protein to sites of active transcription [9, 18]. More recent biochemical studies have implicated the chromodomains as having a role in enzymatic activity and not localization [19]. Genome-wide localization studies in mouse ES cells demonstrated that CHD1 binding correlates with RNA polymerase II (Pol II) and the activating H34Kme3 mark, although bivalent domains enriched in both the H3K4me3 mark and the repressive H3K27me3 mark lack CHD1 [20]. In vitro studies have found that human CHD1 can be targeted to active genes through its interaction with the mediator complex [21]. CHD1 has also been shown to be required for the maintenance of open chromatin and pluripotency of murine embryonic stem cells [20]. In addition, high resolution ChIP-seq experiments revealed that CHD1 is recruited to the promoters of actively transcribed genes, where it evicts the nucleosomes downstream of the promoter allowing for PolII promoter escape [5]. Although CHD1 localizes to regions of active transcription and is generally associated with transcriptional activation, previous studies have indicated that CHD1 plays a negative role in regulating HIV-1 basal transcription and maintaining latency [22, 23].

CHD2 is a member of the CHD1 subfamily, which is characterized by an additional DNA-binding domain located in the C-terminal region [8]. Studies in CHD2 mutant mice suggest possible roles for this protein in development, DNA damage signaling, and maintenance of genome stability [24, 25]. In addition, both CHD1 and CHD2 can regulate deposition of the histone variant H3.3 [26, 27]. Here we show that CHD1 and CHD2 can both regulate HIV-1 gene expression.

## Results

### Isolation of MLV-resistant clones using magnetic cell sorting (MACS)

An insertional mutagenesis screening approach was used to identify novel host factors, which regulate the early stages of retroviral replication. This technique, called SILENCE, uses long terminal repeat (LTR)-encoded cis-acting response elements to induce transcriptional repression in CHO-K1 cells [28]. This system employs a mutagenic MLV vector, CMMP. GFP-NEO-TRE and the *trans*-acting repressor protein tetR-KRAB (TKR), which in the absence of doxycycline, binds to (7 tandem TetO sequences; TRE) located in the long terminal repeat (LTR) region of the mutagenic vector, inducing reversible heterochromatin formation and transcriptional repression within 3 kb of flanking cellular DNA [29, 30]. Since MLV DNA is preferentially integrated close to cellular gene transcription start sites [31], this approach can result in conditional silencing of cellular gene expression.

**Figure 1:**
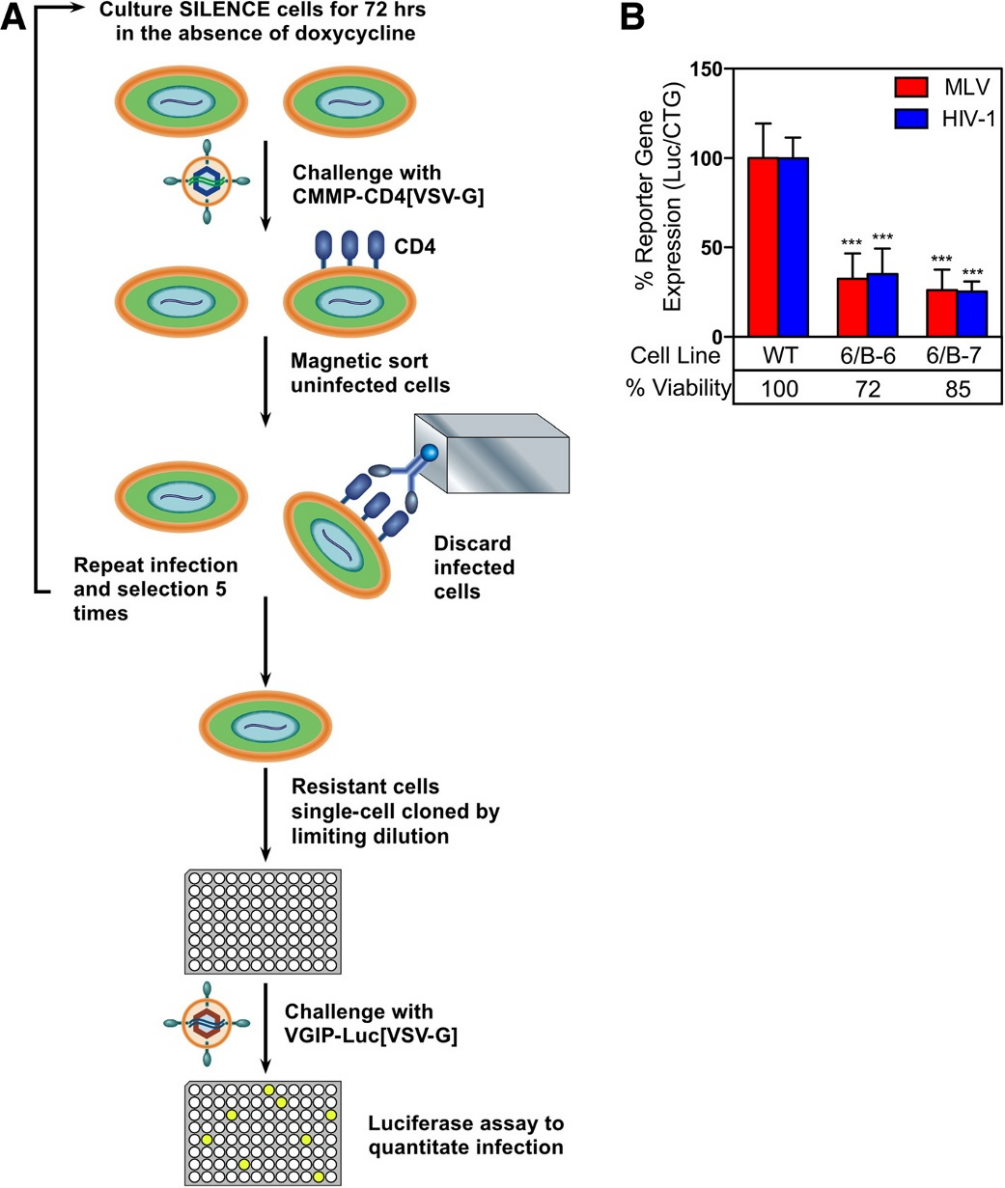
**Isolation and characterization of retrovirus-resistant clones. (A)** Strategy used to isolate MLV-resistant clones. Transcriptional repression of cellular genes flanking the mutagenic vector was induced in ten independent pools of SILENCE cells by withdrawal of doxycycline for 72 h. The cells were then challenged with a VSV-G pseudotyped MLV vector, CMMP-CD4 [VSV-G], encoding human CD4, at a MOI of approximately 1.0 CD4-transducing units. Infected cells were depleted from the population by magnetic separation using an iron-conjugated CD4 antibody. Uninfected cells were allowed to recover in medium containing doxycycline for a minimum of 72 h, prior to a subsequent round of infection and selection. After five rounds of infection and selection, the 6B pool was considered to be relatively resistant to MLV infection, based on the resultant numbers of CD4-expressing cells. Individual cell clones were derived from this pool by limiting dilution and virus resistance was verified by challenging with a VSV-G pseudotyped MLV vector, VGIP3-Luc [VSV-G] encoding firefly luciferase. **(B)** Resistance of two mutant cell clones to infection by MLV and HIV-1 vectors. Wild type CHO-K1 and the 6/B-6 and 6/B-7 mutant CHO-K1 cell clones were challenged with VSV-G pseudotyped vectors encoding firefly luciferase: VGIP3-Luc or NL43 R + E- Luc. Cell viability was also monitored using the CTG assay. The ratios of firefly luciferase activity: cell viability were determined and compared to that of wild type CHO-K1 cells (defined as 100% infection). The data shown is the average mean of three independent experiments each performed with triplicate samples. Error bars indicate the standard deviation. The data was analyzed using an unpaired T-test, ***P value <0.0001.

The SILENCE cells used in this study have been previously described [28]. Ten separate pools of SILENCE cells were cultured for 72 h in the absence of doxycycline in order to repress transcription of host genes near the integrated provirus containing the TREs (Figure 1A). 5 × 10^6^ cells per pool were challenged with replication-defective, VSV-G pseudotyped MLV encoding the human CD4 gene (CMMP-CD4 [VSV-G]), at an MOI of approximately 1.0 for 2 h at 37°C. 28 hpi, infected cells, which express CD4 on their surface, were depleted from the population by magnetic cell sorting (MACS) using an iron-conjugated CD4 antibody. After selection, uninfected cells were allowed to recover in media containing doxycycline, thus allowing for transcription of any silenced genes. After five rounds of selection, one pool of cells (designated as 6/B) was resistant to MLV infection as compared to wild-type CHO-K1 cells (data not shown). This resistant cell population, which exhibited 2.0-fold reduced levels of infectivity compared to wild type CHO-K1 cells, was single cell cloned by limiting dilution and the resulting clones were tested for their susceptibility to infection by a VSV-G pseudotyped MLV vector which encodes luciferase (VGIP-Luc [VSV-G]) (data not shown). Prior to viral challenge, cells were cultured for 72 h in either the presence or absence of doxycycline to determine whether MLV resistance was doxycycline-mediated. At 28 hpi, luciferase activity was measured. Two MLV-resistant cell lines, 6/B-6 and 6/B-7 were identified, although MLV resistance in these clones was not doxycycline-mediated, indicating that disruption of the cellular gene by the mutagenic vector is not dependent on the tetR-KRAB repressor protein. These two cell lines were further characterized and are described below.

### The mutant CHO-K1 cell lines display resistance to infection by both MLV and HIV-1

To determine if the defect in the 6/B-6 and 6/B-7 mutant cell lines was specific to MLV or if it affects HIV-1 infection, wild type CHO-K1 cells and mutant cell lines were seeded in 96 well plates and challenged with either a VSV-G pseudotyped MLV reporter virus VGIP-Luc [VSV-G] or a VSV-G pseudotyped HIV-1 reporter virus pNL43-Luc-R^+^E^−^ [VSV-G] encoding luciferase. At 24 hpi luciferase activity was measured. A luminescent cell viability assay was utilized to monitor viable cell number and to correct for differences in cell number due to variability during plating and different growth rates. Cells lines 6/B-6 and 6/B-7 exhibited a 67% and 65% reduction in MLV reporter gene expression as compared to that seen with wild type CHO-K1 cells (Figure 1B). Viral reporter gene expression following infection with VSV-G pseudotyped HIV-1 reporter virus was reduced to similar levels seen with the VSV-G pseudotyped MLV vectors in both 6/B-6 and 6/B-7 (Figure 1B), indicating that the affected genes in the mutant cell lines are important for both MLV and HIV-1 infection. The mutant cell lines did display a modest growth defect compared to the wild type CHO-K1 cells (Figure 1B), although this defect may be caused by the presence of multiple antibiotics in the media and not the defective gene.

### The Chromodomain-helicase-DNA-binding protein 2 (CHD2) gene is disrupted in the mutant CHO-K1 cell clones

**Figure 2:**
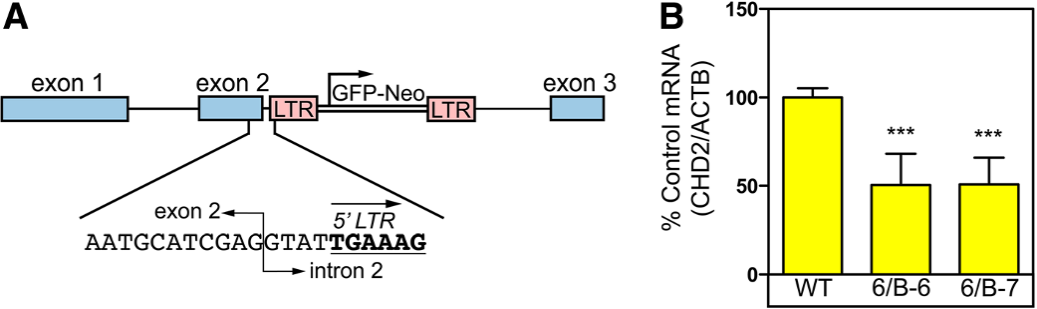
**The mutagenic viral vector is integrated into the CHD2 gene in the mutant cell clones. (A)** The TRE mutagenic viral vector is integrated into intron 2 of the hamster CHD2 gene. The DNA sequence around the integration site is expanded to show the exon2/intron2 boundary and the adjacent viral DNA (bold and underlined). **(B)** Quantitative RT-PCR was used to measure CHD2 mRNA levels relative to control β-actin mRNA levels in wild type and mutant cells. The ratios of CHD2 mRNA: β-actin mRNA were determined and compared to wild type CHO-K1 cells (defined as 100% Control). The data shown is the average mean of three independent experiments. Error bars indicate the standard deviation. The data was analyzed using an unpaired T-test, ***P value <0.0001.

Southern blot analysis revealed that the 6B-6 and 6B-7 cell clones each contained a single mutagenic CMMP. GFP-NEO-TRE provirus (data not shown). We then employed Inverse PCR to determine the integration site of the mutagenic CMMP. GFP-NEO-TRE virus in the 6/B-6 and 6/B-7 cell lines as described in the materials and methods section. This analysis revealed that the mutagenic viral vector was integrated near the 5’-end of intron 2 of the Chromodomain-helicase-DNA-binding protein 2 (CHD2) gene in both cell lines (Figure 2A), therefore the two cell clones were derived from the same virus-resistant cell in the 6B cell population. Consistent with gene disruption, CHD2 mRNA levels were decreased by approximately 50% in cell clones 6B-6 and 6B-7 relative to wild-type CHO-K1 cells (Figure 2B). These data suggest that disruption of the CHD2 gene may confer resistance to retroviral infection in CHO-K1 cells.

### CHD2 and the related CHD1 protein positively regulate retrovirus infection in human cells

To determine if CHD2 also positively regulates retroviral infection in human cells, both loss-of-function and gain-of-function experiments were performed in HEK293T cells. This cell line was chosen for this study because of its high efficiency of RNAi-knockdown and cDNA overexpression. We also included the highly related CHD1 protein in this analysis since CHD2 is a member of the CHD1 subfamily of CHD proteins [11].

**Figure 3:**
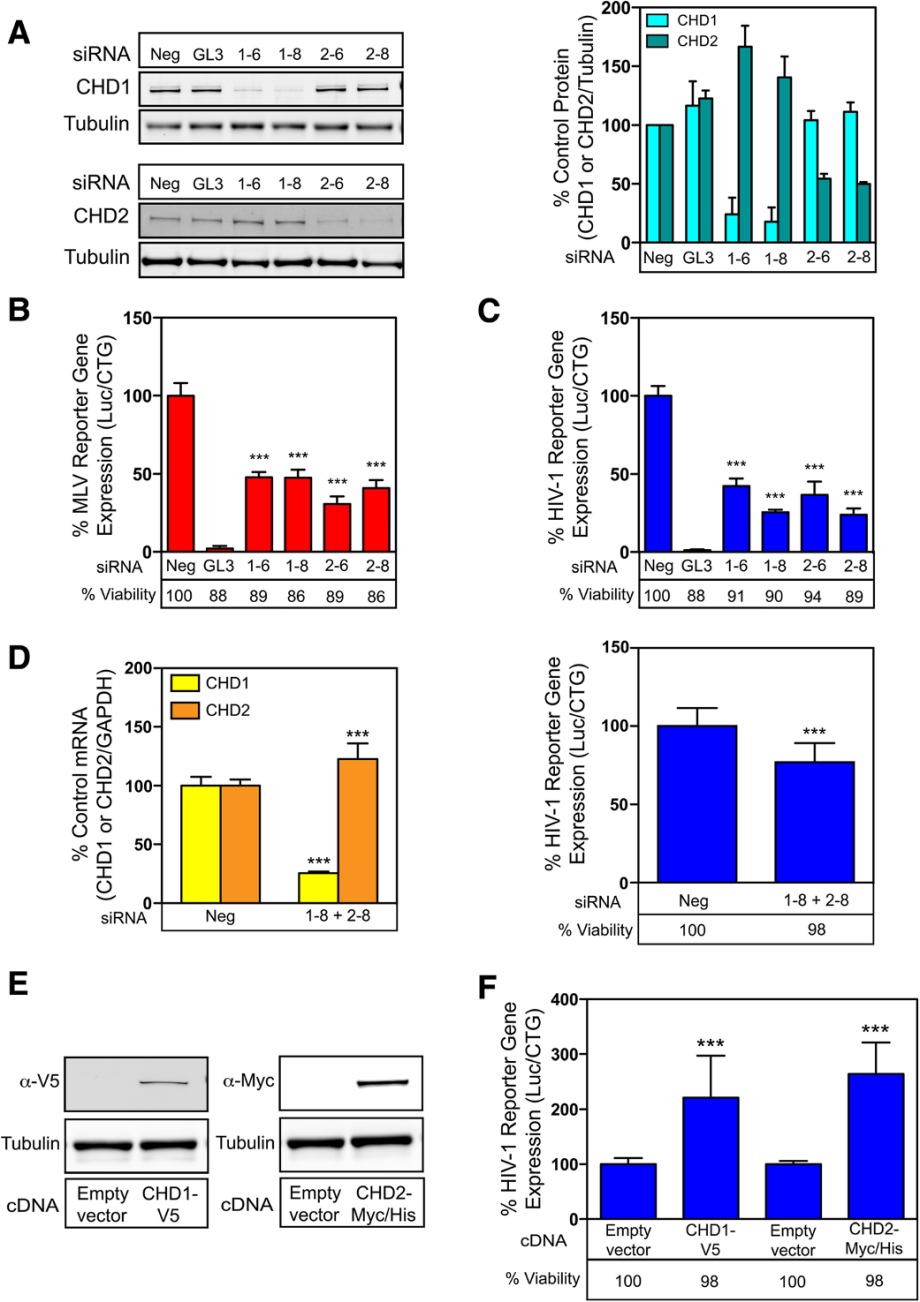
**CHD2 and CHD1 regulate retroviral infection in human cells. (A)** siRNAs targeting CHD1 and CHD2 were transfected into HEK293T cells. A non-targeting siRNA (Neg) and a siRNA targeting the virus-encoded luciferase (GL3) were used as controls. (Left panel) Immunoblot analysis of CHD1, CHD2 and tubulin protein levels at 48 hours post-transfection. (Right panel) Protein levels were quantified using the Licor Odyssey infrared imaging system and the ratios of CHD1 or CHD2 protein:tubulin were determined and compared to the non-targeting control (defined as 100% Control). The data shown is the average mean of three independent experiments. **(B)** HEK293T cells transfected with the siRNAs were challenged with the MLV vector or **(C)** the HIV-1 vector. The ratios of luciferase activity:cell viability were determined and compared to the non-targeting control (defined as 100% infection). **(D)** siRNAs targeting both CHD1 and CHD2, or a control siRNA (Neg) were transfected into SupT1 cells. (Right panel) The cells were challenged with the HIV-1 vector as described above. (Left panel) CHD1 and CHD2 mRNA levels were measured by qRT-PCR. The ratios of CHD1 or CHD2 mRNA:GAPDH mRNA were determined and compared to the non-targeting control (defined as 100% Control). (Right panel) The ratio of virus-encoded luciferase activity:viable cell number was determined and compared to the non-targeting control (defined as 100% infection). **(E)** Immunoblot analysis of CHD1-V5, CHD2-Myc/His and tubulin protein levels at 48 hours post-transfection in transfected HEK293T cells. **(F)** The cDNA-expressing cells were challenged with the HIV-1 vector at 48 hours post-transfection. The ratio of virus-encoded luciferase activity:viable cell number was determined and compared to the non-targeting control (defined as 100% infection). The data shown is the average mean of three independent experiments each performed with triplicate samples. Error bars indicate the standard deviation. The data was analyzed using an unpaired T-test, ***P value <0.0001.

RNAi-knockdown of CHD1 and CHD2 protein levels was achieved with 2 independent siRNAs targeting each gene (Figure 3A) and without any overt cell toxicity (Figure 3B and 3C). The siRNA transfected cells were challenged with the VSV-G pseudotyped VGIP-Luc MLV vector, or the pNL43-Luc-R^+^E^−^ HIV-1 vector, and virus-encoded luciferase activity was measured. Depletion of either CHD1 or CHD2 had a significant impact on MLV and HIV reporter gene expression as compared to a negative control siRNA (Figure 3B and 3C). We also reproducibly noted that RNAi-knockdown of CHD1 led to a modest increase in the level of CHD2 protein (Figure 3A), an effect that was also seen at the mRNA level (data not shown).

The siRNAs targeting both CHD-1 and CHD-2 also led to a modest but highly reproducible and statistically significant decrease in HIV-1 reporter gene expression in human SupT1 cells, a T-cell line that is more physiologically relevant for HIV-1 infection (Figure 3D, right panel). Reduced infection in this case was associated with a decrease in CHD1 mRNA levels, as CHD2 mRNA was not depleted in these experiments (Figure 3D, left panel).

In the gain-of-function studies, epitope tagged versions of CHD1 or CHD2 (V5-tagged CHD1 or Myc/His-tagged CHD2) were expressed in HEK293T cells (Figure 3E). These cells were challenged with the VSV-G pseudotyped pNL43-Luc-R^+^E^−^ HIV-1 reporter virus and infection was measured by virus-encoded firefly luciferase as before. The efficiency of HIV-1 reporter gene expression was significantly enhanced in cells that overexpressed either epitope tagged-CHD1 or -CHD2, compared to the empty plasmid vector controls (Figure 3F). Cell viability was not affected under these conditions. Taken together, these results demonstrate that CHD1 and CHD2 are positive regulators of HIV-1 gene expression in human cells.

### The effect of CHD1 and CHD2 in HEK293T cells maps to HIV-1 transcription

**Figure 4:**
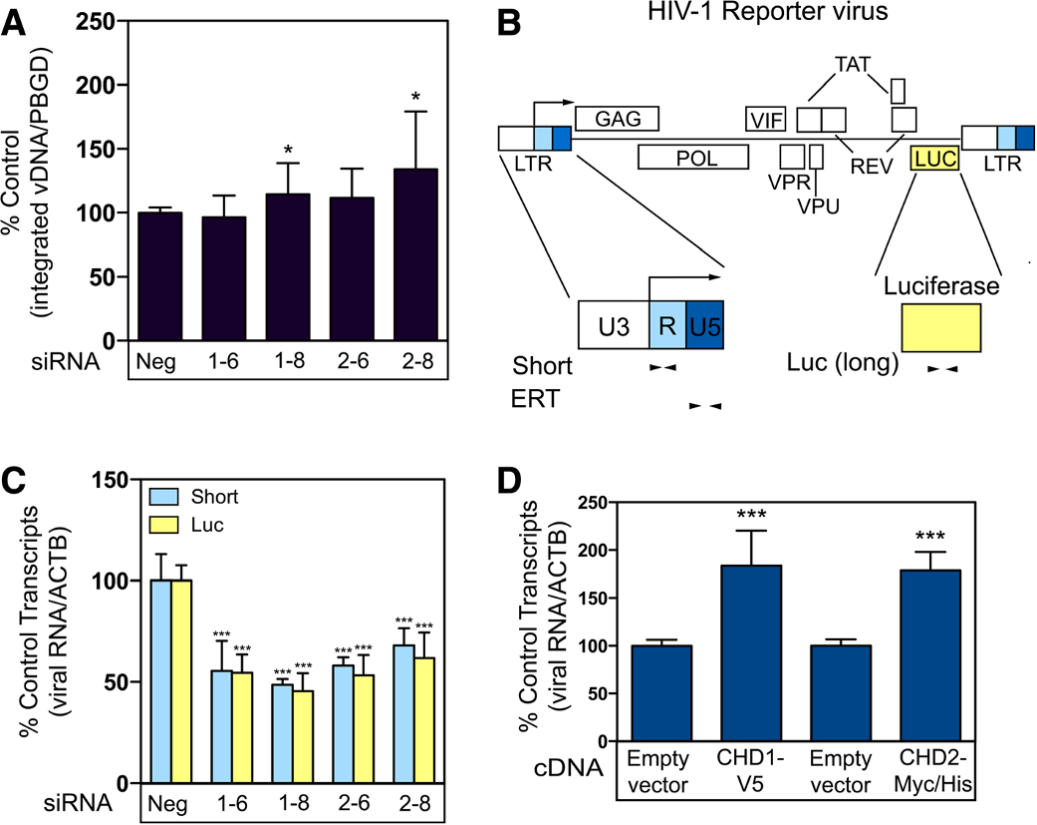
**CHD1 and CHD2 positively regulate HIV-1 transcription. (A)** siRNAs targeting CHD1 and CHD2, or a control siRNA (Neg) were reverse transfected into HEK293T cells. 48 hours post-transfection the cells were challenged with the VSV-G pseudotyped HIV-1 viral vector NL43 R + E- Luc and incubated for 7 days to eliminate extrachromosomal HIV-1 DNA. qPCR analysis was performed to measure the level of integrated viral DNA. The ratio of viral DNA: PBGD control was determined and compared to the non-targeting control (defined as 100% control). **(B)** Schematic of the HIV-1 reporter virus and the location of the oligonucleotide primers used for qRT-PCR amplification of viral RNA transcripts. The short primer set amplifies the first 59 nucleotides of the HIV transcript and will amplify both the short abortive transcripts and the full-length transcripts. The luciferase primers will amplify only elongated viral transcripts. **(C)** Measurement of short and long HIV-1 transcripts at 24 hpi in cells transfected with siRNAs targeting CHD1 or CHD2 and challenged with the VSV-G pseudotyped HIV viral vector NL43 R + E- Luc. The HIV-1 RNA levels were measured by qRT-PCR and the ratios of HIV RNA: β-actin mRNA were determined and compared to the non-targeting control siRNA (defined as 100% Control). **(D)** HEK293T cells were transfected with plasmids encoding epitope-tagged CHD1 or CHD2 proteins and challenged with the VSV-G pseudotyped HIV viral vector NL43 R + E- Luc. The HIV-1 RNA levels were measured by qRT-PCR as above using the ERT primers. The ratios of HIV RNA: β-actin mRNA were determined and compared to the empty vector controls (defined as 100% Control). The data shown is the average mean of three independent experiments. Error bars indicate the standard deviation. The data was analyzed using an unpaired T-test, *P value <0.01, ***P value <0.0001.

Real-time quantitative PCR analysis revealed that RNAi-knockdown of either CHD1 or CHD2 in HEK293T cells did not impact the levels of reverse transcribed HIV-1 DNA (data not shown) or HIV-1 proviral DNA (Figure 4A). However, real-time quantitative PCR analysis revealed a significant reduction in all HIV-1 mRNA transcripts in cells transfected with either CHD1 or CHD2 siRNAs indicating a role early in the transcription cycle (Figure 4C). Although we did not observe the characteristic increase in the short abortive transcripts associated with a defect in elongation, these results do not preclude the possibility of a role in elongation or other downstream steps in the transcription cycle. Consistently, we detected an increase in the levels of HIV-1 mRNA transcripts in cells transfected with the epitope-tagged CHD1 or CHD2 proteins (Figure 4D). Taken together, these data are consistent with a role for CHD1 and CHD2 as positive regulators of HIV-1 transcription.

## Discussion

In this report we provide genetic evidence that the chromodomain proteins CHD1 and CHD2 are positive regulators of retroviral replication. Firstly, an insertional mutagenesis screen identified CHD2 as the disrupted gene in mutant CHO-K1 cells that were resistant to MLV and HIV-1 infection. Secondly, gain-of-function and loss-of-function experiments in HEK293T cells confirmed that CHD2 and the highly related CHD1 protein positively regulate MLV and HIV-1 infection. Thirdly, siRNAs targeting CHD1 reduced HIV-1 infection of human SupT1 T cells. The effect of these two chromodomain proteins was mapped to a stage in transcription, which precedes elongation, indicating that CHD1 and CHD2 play a role early in the transcription cycle. The effect of these proteins on virus replication is consistent with their known roles in the cell. Different members of the CHD family generally function at different steps during cellular gene transcription and can act as either transcriptional activators or repressors [32]. CHD1 localizes to H3K4me3 positive promoters [20] and to regions of active transcription [15], and is associated with transcriptional activation.

In contrast to our findings, studies that used a chimeric yeast-HIV transcription model, implicated CHD1 as a repressor of HIV-1 basal transcription [22]. The difference between these findings and our result could be due to the choice of experimental model systems. While the yeast CHD1 protein’s main role during transcription is reassembly and repositioning of nucleosomes in the gene body during elongation, the mammalian CHD1 also functions at the promoter where it promotes nucleosome destabilization in order to overcome the nucleosome barrier to Pol II promoter escape [5, 6, 33]. In addition, unlike the mammalian protein, the yeast CHD1 chromodomains lack the key residues required for binding to methylated H3K4 [9, 18], which is predicted to influence both localization and activity [19]. CHD1 has also been linked to the maintenance of HIV latency via transcriptional interference in two J-Lat clones E27 and A2, which harbor a HIV minigenome within introns of highly active cellular genes [23]. In the transcriptional interference model of virus latency it has been proposed that CHD1 and other chromatin assembly factors associated with transcriptional elongation can exert their repressive effects by assembling chromatin into a closed repressive configuration thereby silencing the HIV promoter [23]. CHD1 does have what appears to be opposing activities at different steps during the transcription cycle, which could account for the different results seen in our study. Early in the transcription cycle, CHD1 promotes nucleosome destabilization at promoters, resulting in efficient Pol II promoter escape and downstream progression; while during elongation, CHD1 promotes nucleosome stabilization and inhibits cryptic transcription [5, 6]. While the relationship of these findings, obtained with proviruses that are also subject to negative regulation by a highly active cellular gene, and our results with cells newly infected with MLV or HIV-1 is not yet clear, it is important to note that our findings are consistent with the established role of the CHD1 subfamily of proteins as activators of gene transcription.

Possible models of CHD1 and CHD2 regulation of retroviral gene expression include histone modification and/or nucleosome remodeling events. In addition, CHD1 and CHD2 may affect other factors needed for HIV transcription either by recruiting factors to the HIV LTR or by acting at the promoters of the required host gene. It is also possible that these cellular proteins might influence HIV-1 DNA integration site specificity, which in turn could influence the efficiency of viral transcription. Future experiments will be aimed at determining precisely how these two proteins regulate HIV-1 gene expression.

## Conclusions

In this study, we employed an insertional mutagenesis screening technique in CHO-K1 to identify novel cellular regulators of retroviral infection. This technique initially identified the ATP-dependent chromatin remodeler, CHD2, as a positive regulator of both MLV and HIV-1 replication. Gain-of-function and loss-of-function experiments performed in human cells confirmed that CHD2 and the highly related CHD1 protein positively regulate MLV and HIV-1 infection. The requirement for CHD1 or CHD2 was more specifically mapped to an early stage of viral transcription, which is consistent with their known roles in the cell. These results demonstrate that both CHD1 and CHD2 are important positive regulators of HIV-1 gene expression. Future studies will provide a more detailed view of the role of CHD1 and CHD2 in the transcription cycle of HIV-1 infection and will further contribute to our understanding of epigenetic regulation of transcription during HIV infection.

## Materials and methods

### Ethics statement

No animals or human subjects were used in this study.

### Plasmids

The viral genome plasmids pCMMP-CD4, pVGIP3 and pNL43-Luc-R^+^E^−^ have been described previously [7, 34, 35]. The CHD1 Gateway entry clone (HsCD00365806) was acquired from Dana-Farber/ Harvard Cancer Center DNA Resource Core. The CHD1-V5 expression clone was generated by transferring the CHD1 cDNA from the entry clone to the Gateway pcDNA6.2/V5-DEST (Invitrogen) destination vector by LR recombination. The CHD2 cDNA was synthesized from total RNA isolated from 293 T cells using the SuperScript III First Strand Synthesis Kit (Invitrogen) and the gene specific primer CHD2(CD)-R1 (Table 1). The CHD2-Myc/His expression clone was generated by PCR amplification of the coding sequence from CHD2 cDNA using the primers CHD2-XhoI-F3 and CHD2-BamHI-R6 (Table 1). PCR products were digested with XhoI and BamHI and cloned into pCDNA3.1-Myc/His (Invitrogen).

**Table 1:** **Primer and Probe sequences**

Name	Sequence 5'-3'
CHD2(CD)-R1	CTTTATGTTTTCCGAACATTCCAGTTATAATCTGG
CHD2-XhoI-F3	AGTCAGCTCGAGATGATGAGAAATAAGGACAAAAG
CHD2-BamHI-R6	AGTCACGGATCCCTTAATGTTTTCCGAACATTCCAG
iPCR1	GAGAAGTTCAGATCAAGGTTAGGAACAGAG
iPCR2	GGTAGGAGACGAGAACCTAAAACAGTTCCCGCC
iPCR3	GTTATGTATTTTCCATGCCTTGCAAAATGGC
iPCR4	GCTTTCGGTTTGGGACCGAAGCCGCGCCG
MH531	TGTGTGCCCGTCTGTTGTGT
MH532V	GAGTCCTGCGTCGAGAGATC
LRT-Probe	CAGTGGCGCCCGAACAGGGA
PBGD1	AAGGGATTCACTCAGGCTCTTTC
PBGD2	GGCATGTTCAAGCTCCTTGG
PBGD-Probe	CCGGCAGATTGGAGAGAAAAGCCTGT
Lucif 1	TTGGGCGCGTTATTTATCG
Lucif 2	GCAATTCACGTTCATTATAAATGTCG
HIV Start	GGGTCTCTCTGGTTAGA
HIV Short 3"	GGGTTCCCTAGTTAGCC
ERT2F	GTGCCCGTCTGTTGTGTGAC
ERT2R	GGCGCCACTGCTAGAGATTT
CHD1-F	GATGAAGATTGGCAAATGTCTG
CHD1-R	ATTTTGAGGTTTTCTGCTTTTG
CHD2-F3	CGAAAACAGGCACTGGACCACT
CHD2-R3	GATGACGACTGTGTCCGCTGAA
ACTB-F	CCTGGCACCCAGCACAAT
ACTB-R	GCCGATCCACACGGAGTACT
GAPDH-F2	CATGAGAAGTATGACAACAGCCT
GAPDH-R2	AGTCCTTCCACGATACCAAAGT
CgCHD2-F3	CGAAAGCAGGCATTGGATCACT
CgCHD2-R3	GATGACAACTGTGTCCGCTGAA
CgACTB-F1	GGATGCAGAAGGAGATCACT
CgACTB-R1	GAGTACTTGCGCTCAGGAGGA

### Cell culture and virus production

Chinese hamster ovary CHO-K1 cells were cultured in F-12 media (Invitrogen) supplemented with 10% FBS (Hyclone). Human embryonic kidney 293 T cells were cultured in DMEM (Invitrogen) supplemented with 10% FBS (Hyclone). The human CD4+ T-cell line SupT1 was cultured in RPMI (Invitrogen) supplemented with 10% FBS (Hyclone). SILENCE cells [28] were provided by Dr. Kenneth A. Bradley and were cultured as described above for CHO-K1 cells with the addition of 900 μg/mL G418 (Gibco), 10 μg/mL puromycin (Gibco), and 1 μg/mL doxycycline (Sigma).

VSV-G pseudotyped MLV virus was generated by cotransfection of 293 T cells with the appropriate viral genome plasmid, pMD.gagpol encoding MLV Gag/Pol [33] and pCMV-VSV-G encoding the VSV envelope glycoprotein [36]. 48 h post-transfection, retroviral supernatant was harvested, filtered, and stored at −80°C, as described previously [2]. VSV-G pseudotyped HIV-1 luciferase reporter virus was generated by cotransfection of 293 T cells with pNL43-Luc-R^+^E^−^ and pCMV-VSV-G [36]. 48 h post-transfection, retroviral supernatant was harvested, filtered, and treated with DNaseI (Roche) to remove contaminating plasmid DNA. The virus was then aliquoted and stored at −80°C, as described previously [2].

### Isolation of MLV-resistant CHO-K1 cells

Ten separate pools, each consisting of 5 × 10^6^ SILENCE cells [28] were cultured for 72 h in the absence of doxycycline, followed by infection with CMMP-CD4 [VSV-G] at an approximate MOI 1.0 CD4 transducing units for 2 h at 37°C in the presence of 4 μg/ml Polybrene. After 2 h, virus-containing media was removed and replaced with fresh media. At 28 h post-infection (hpi), cells were detached from plates with 5 mM EDTA/Dulbecco's phosphate-buffered saline (DPBS) and washed with cold DPBS. Cells were resuspended in PBE (DPBS supplemented with 1% BSA and 2 mM EDTA) and incubated with human CD4 microbeads (Miltenyi Biotec Inc.) for 15 min at 4°C. Large cell (LC) columns (Miltenyi Biotec Inc.) were placed in the OctoMACS magnetic separator (Miltenyi Biotec Inc.) and washed with 1 ml of PBE. Cell suspensions were filtered through a 30 μm MACS pre-separation filters (Miltenyi Biotec Inc.) before being applied to the pre-washed LC columns. Cells were washed three times with 0.5 ml of PBE. The flow through and washes were collected and the cells were pelleted and plated in medium containing doxycycline. Cells were expanded and allowed to recover for a minimum of 72 h before the next viral challenge. The infection and selections were repeated as before for a total of five times at which time there was observable resistance to MLV infection in mutant cell pool 6/B compared to wild type CHO-K1 cells. The resistant 6/B pool was then subjected to single cell cloning by limiting dilution in 96-well plates. In order to identify MLV-resistant clones, single cell clones from pool 6/B were plated in replicate 96-well plates and one plate was challenged with VGIP3-Luc [VSV-G] in the presence of 4 μg/ml Polybrene. At 28 hpi, luciferase activity was quantitated with the BrightGlo luciferase assay (Promega) and the second plate was assayed for viable cell number using the CellTiter-Glo cell viability assay (Promega).

### Quantitative MLV and HIV Infectivity assays (CHO-K1 cells)

For each cell line tested, 1 × 10^4^ cells were seeded in 9 wells of a black 96 well plate. The following day cells were infected with either VGIP3-Luc [VSV-G] or NL43-Luc-R^+^E^−^ [VSV-G] in the presence of 4 μg/ml Polybrene in triplicate wells. 24 hpi, luciferase activity was quantitated with the BrightGlo luciferase assay (Promega) and the uninfected wells were assayed for viable cell number using the CellTiter-Glo cell viability assay (Promega). Luciferase results were normalized to cell number.

### Nested inverse PCR

Genomic DNA (gDNA) was isolated using the PURElink genomic DNA mini kit (Invitrogen, Carlsbad, CA.). 2 μg of gDNA was digested overnight with PstI in a total volume of 40 μl. The following day the restriction enzyme was heat-inactivated for 20 min at 65°C and 20 μl of the sample was used in a ligation reaction with T4-ligase (NEB) in a final volume of 1 ml and incubated overnight at room temperature. The DNA was phenol/chloroform extracted and resuspended in 50 μl of H_2_O. The first round of PCR was performed using 2 μl of ligated-DNA as template with MLV-specific primers iPCR1 and iPCR2 (Table 1). The second nested round of PCR was performed with 2 μl of the first PCR reaction with the primers iPCR3 and iPCR4 (Table 1). The PCR products were then cloned into the pCR®-Blunt II-TOPO® Vector (Invitrogen) and sequenced.

### RNAi experiments

The small interfering RNAs (siRNAs) used in these experiments were human CHD1 1–6 (On-TARGETplus CHD1 si-6, Dharmacon), CHD1 1–8 (On-TARGETplus CHD1 si-8, Dharmacon), CHD2 2–6 (On-TARGETplus CHD2 si-6, Dharmacon), CHD2 2–8 (On-TARGETplus CHD2 si-8, Dharmacon) ON-TARGETplus Non-targeting siRNA #1 (Dharmacon) and Luciferase GL3 (Qiagen). siRNAs were reverse transfected into HEK293T cells in either 96 (x 3 replicates) or 48 well plates using the Lipofectamine RNAiMAX transfection reagent (Invitrogen). siRNAs and Lipofectamine RNAiMAX were diluted separately into Optimem (Invitrogen), diluted siRNAs were then added to wells followed by the addition diluted Lipofectamine RNAiMAX. After 20 min, aliquots of HEK293T cells freshly resuspended in DMEM supplemented with 20% FBS (Invitrogen) were added to each well. For chemiluminescent infectivity assay, cells were infected 48 h post-transfection with either VGIP3-Luc [VSV-G] or NL43-Luc-R^+^E^−^ [VSV-G] in triplicate wells of a 96 well plate. 24 hpi, luciferase activity was quantitated with the BrightGlo luciferase assay (Promega) and the uninfected wells were assayed for viable cell number using the CellTiter-Glo cell viability assay (Promega). Luciferase results were normalized to cell number.

siRNAs were transfected into SupT1 cells using the Neon Transfection System (Invitrogen) according to the manufacturer’s protocol. Briefly cells were resuspended at a concentration of 2 × 10^7^ cells/ml in 10 μl Buffer R and mixed with siRNAs. Cells were electroporated using protocol number #24 (1600 (V), 10 (pulse width), 3 (pulse #) and then transferred to the well of a 24-well plate containing 400 μl of complete RPMI media. 48 h post-transfection 600 μl of fresh complete RPMI media was added to each well and 100 μl of cells were aliquoted into two 96-well plates (x 3 replicates). Cells were then infected with NL43-Luc-R^+^E^−^ [VSV-G] or mock-infected with media in triplicate wells and spinoculated for 90 min at 1200 × g at 25°C. At 24 hpi, luciferase activity was quantitated with the BrightGlo luciferase assay (Promega) and the mock-infected wells were assayed for viable cell number using the CellTiter-Glo cell viability assay (Promega). Viral reporter luciferase results were normalized to cell number.

### Viral DNA and RNA quantitation by real-time qPCR

To measure proviral DNA content in infected cells, HEK293T cells were reverse transfected in 48 well plates with siRNAs as described above. 48 h post-transfection cells were infected with DNAase I treated NL43-Luc-R^+^E^−^ [VSV-G]. 24 hpi, the virus containing media was removed and the cells were dislodged from each well and transferred to 6 well plates. At 7 days post-infection (dpi) genomic DNA was isolated using the PURElink genomic DNA mini kit (Invitrogen, Carlsbad, CA.). qPCR reactions were performed using 30 ng of genomic DNA and the primer-probe sets; MH531, MH532V, probe LRT-P [2, 37] for quantitating viral DNA, and PBGD1, PBGD2 and probe PBGD-P [2] for the cellular gene porphobilinogen deaminase (PBGD) (Table 1). Real-time PCR assays were performed in triplicate using TaqMan Fast Universal PCR Master Mix (Applied Biosystems). The number of copies in each reaction was quantified using a standard curve produced by amplification of plasmid DNA containing the appropriate target sequence. Proviral DNA levels were normalized to PBGD copy number.

To measure HIV RNA transcript levels in infected HEK293T cells were reverse transfected in 48 well plates with siRNAs as described above. 48 h post-transfection cells were infected with DNAase I treated NL43-Luc-R^+^E^−^ [VSV-G]. 24 hpi total RNA was isolated using the miRNeasy Mini Kit (Qiagen). cDNA synthesis was performed using the QuantiTech Reverse Transcription Kit (Qiagen). The following primer sets were used for Real-time qPCR: for short HIV transcripts; HIV Start and HIV Short 3’ [38]; for HIV full-length transcripts; Lucif1 and Lucif2; for Beta-actin; ACTB-F and ACTB-R. Real-time PCR assays were performed in triplicate using Fast Sybr Green Master Mix (Applied Biosystems). The number of copies in each reaction was quantified using a standard curve produced by amplification of plasmid DNA containing the appropriate target sequence. HIV RNA transcripts were normalized to ACTB copy number.

### Immunoblotting

The antibodies used for immunoblot analysis were CHD1 (Bethyl Laboratories), CHD2 (Cell Signaling), α/β-Tubulin (Cell Signaling) and Alexa Fluor 680 donkey anti-rabbit IgG (Invitrogen). 48 h post-transfection cells were lysed in 2x NuPAGE LDS sample buffer (Invitrogen) and resolved by SDS-PAGE on NuPAGE 4-12% Bis-Tris gels (Invitrogen). Proteins were transferred to Immobilon-FL PVDF membranes (Millipore) and membranes were for blocked for 1 h in 0.1% Casein/0.2x PBS (Biorad). The membrane was then probed with primary antibody in blocking buffer +0.1% Tween-20 overnight at 4°C. Membranes were washed three times with PBS +0.1% Tween-20, then incubated with Alexa Fluor 680 donkey anti-rabbit secondary antibody in blocking buffer +0.1% Tween-20 + 0.01% SDS for 1 h at room temperature. Immunoblots were washed, then visualized and quantitated using an Odyssey infrared imaging system (Licor). CHD1 and CHD2 protein levels were normalized to α/β-Tubulin levels.

### mRNA quantitation by real-time quantitative PCR

Total RNA was isolated using the RNeasy Mini kit (Qiagen). cDNA synthesis was performed using the QuantiTech Reverse Transcription Kit (Qiagen). For RNAi experiments, total RNA was isolated 48 h post-transfection. The following primer sets were used for Real-time qPCR: for CHD2 in CHO-K1 cells; CgCHD2-F3 and CgCHD2-R3; for β-Actin in CHO-K1 cells; CgACTB-F1 and CgACTB-R1; for CHD1 in human cells; CHD1-F and CHD1-R; for CHD2 in human cells; CHD2-F3 and CHD2-R3; for β-Actin in 293 T cells; ACTB-F and ACTB-R; for GAPDH in SupT1 cells; GAPDH-F2 and GAPDH-R2 (Table 1).

### Transient transfection assays

Plasmid DNA was transfected into HEK293T cells in either 96 or 48 well plates using the Transit LT-1 transfection reagent (Mirus). For chemiluminescent infectivity assays, cells were infected 48 h post-transfection NL43-Luc-R^+^E^−^ [VSV-G] in triplicate wells of a 96 well plate. At 24 hpi, luciferase activity was quantitated with the BrightGlo luciferase assay (Promega) and the uninfected wells were assayed for viable cell number using the CellTiter-Glo cell viability assay (Promega,). Luciferase results were normalized to cell number. To measure HIV RNA transcript levels, HEK293T cells were transfected with plasmid DNA in 48 well plates. 48 h post-transfection cells were infected with DNAase I treated NL43-Luc-R^+^E^−^ [VSV-G]. At 24 hpi total RNA was collected and analyzed as described previously for siRNA experiments, using the primer sets; ERT2F and ERT2R for viral RNA transcripts [39] and ACTB-F and ACTB-R for Beta-actin (Table 1).
